# Synthetic distracted driving (SynDD1) dataset for analyzing distracted behaviors and various gaze zones of a driver

**DOI:** 10.1016/j.dib.2022.108793

**Published:** 2022-11-29

**Authors:** Mohammed Shaiqur Rahman, Archana Venkatachalapathy, Anuj Sharma, Jiyang Wang, Senem Velipasalar Gursoy, David Anastasiu, Shuo Wang

**Affiliations:** a Iowa State University, Ames, Iowa, USA; b Syracuse University, Syracuse, New York, USA; c Santa Clara University, California, USA; d NVIDIA Corporation, California, USA

**Keywords:** Driver behavior, Driver distraction, Head orientation, Activity recognition, Activity analysis

## Abstract

This article presents a synthetic distracted driving (SynDD1) dataset for machine learning models to detect and analyze drivers' various distracted behavior and different gaze zones. We collected the data in a stationary vehicle using three in-vehicle cameras positioned at locations: on the dashboard, near the rearview mirror, and on the top right-side window corner. The dataset contains two activity types: distracted activities [1], [2], [3], and gaze zones [4], [5], [6] for each participant and each activity type has two sets: without appearance blocks and with appearance blocks, such as wearing a hat or sunglasses. The order and duration of each activity for each participant are random. In addition, the dataset contains manual annotations for each activity, having its start and end time annotated. Researchers could use this dataset to evaluate the performance of machine learning algorithms for the classification of various distracting activities and gaze zones of drivers.

**Specifications Table**

**Table:** 

Subject	Data Science
Specific subject area	Driver behavior analysis, Driver safety
Type of data	InfraRed Videos, Annotation files
How the data were acquired	Three in-vehicle cameras acquired data. We requested the participants to sit in the driver's seat and then instructed them to perform driver distracting activities or gaze at some region for a short time interval. The instructions were given either by a person sitting in the backseat or played on a portable audio player. Instruments: Kingslim D1 dash cam [7]
Data format	Video files are .MP4 format and annotation files are .csv files
Description of data collection	We designed a survey using a Qualtrics form and selected the respondents based on the criteria that created a balanced representation by gender, age, and ethnicity.
Data source location	• Institution: Iowa State University • City/Town/Region: Ames, Iowa • Country: USA • Latitude 42.0267° N, Longitude 93.6465° W
Data accessibility	Repository name: Synthetic Distracted Driving (SynDD1) Dataset Data identification number: 10.17632/ptcp7rp3wb.4 Direct URL to data: https://data.mendeley.com/datasets/ptcp7rp3wb/4

## Value of the Data

• The data will serve as baseline data for the training, testing, and validation of computer vision-based machine learning models having the primary objective of detecting and classifying driver behaviors and gaze zones.
• The data can be used to benchmark the performance of various machine learning models designed with a similar objective.
• The data can be used by researchers working on analyzing driver behaviors where their objective is detection and classification of driver activities.
• The data can help researchers design and build a driver-assist system that would improve drivers' safety on the road by alerting them during driving.

## 1 Data Description

**Fig. 1:**
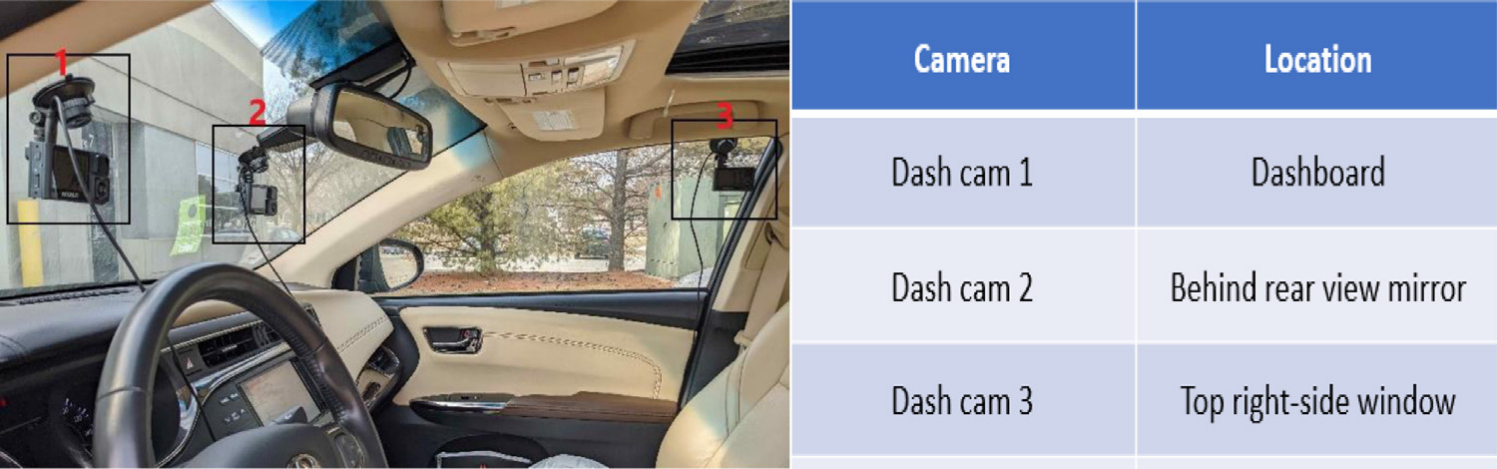
Showing camera positions inside car

We annotated the data for each participant for all the camera views, as shown in Fig. 1. The annotation files(.csv) contains information as shown in Table 1. The duration of each activity is up to 24 seconds, and for different participants, the order of activities is different.

**Table 1:** Showing variables in the data set.

User ID	Participant identification number
Filename	Video file name
Camera View	Camera positioned near the dashboard, rearview mirror, or right-side window
Activity Type	Gaze or distracted
Start Time	Start time (h: mm: ss) of activity (may include up to 10 seconds before the actual activity starts)
End Time	End time (h: mm: ss) of activity
Label	Various activities performed by the participant; see Tables 7 and 8 for more details
Appearance Block	Participants may be wearing hats or sunglass or none

The dash cam we used for data collection has the specification as shown in Table 2, and the data acquisition requirements are shown in Table 3.

**Table 2:** Showing Specification of video acquisition system.

Camera model	Kingslim D1 Pro Dual Dash Cam
Resolution	1920 × 1080P
Frame rate	30fps
Sensor	1/ (2.8)” SONY IMX307 industrial grade
Aperture	f/1.8 large
Lens angle	170-degree wide angle
LED	4 IR LED's
Pixel size	2.9μ x 2.9μ

**Table 3:** Showing Data acquisition requirement.

Requirement	Description
Data Recording	Video duration: 300 s multiple videos
	Frequency: 50hz
	Data file format: MP4
Data Storing	SD card reader: Sandisk micro
Operating system	Windows 10 (& above), Mac OS Sierra (& above)
Communication	USB 2, USB C

For each participant, there are twelve video files. Each camera has two activity types (gaze/distracted), and each type has two sets (with/without appearance block), as shown in Table 4. The videos are infra-red, and we removed the audio from the video files.

**Table 4:** Showing different videos for a camera.

Dashboard	Gaze	Without appearance block
		With appearance block
	Distracted	Without appearance block
		With appearance block

## 2 Experimental Design, Materials and Methods

The synthetic data collection process involved three in-vehicle cameras [7] positioned near the dashboard, rearview mirror, and top corner of the right-side window, as shown in Fig. 1. We requested the participants to sit inside a stationary vehicle in the driver's seat. Then we gave them instructions to gaze at a particular region or perform a distracting activity continuously for a short time interval. The dataset, thus generated, we call it as Synthetic Distracted Driving (SynDD1).

SynDD1 details are shown in Table 5, and the specification of the videos in the dataset are shown in Table 6.

**Table 5:** Showing dataset details [9].

Title	Synthetic Distracted Driving (SynDD1) Dataset
Description	Synthetic dataset for machine learning models to detect and analyze drivers' various distracted behavior and different gaze zones.
Identifier	https://data.mendeley.com/datasets/ptcp7rp3wb/4
License	DATASET LICENSE AGREEMENT.pdf (available in the dataset)
Modified	2 November 2022
File formats	MP4, csv
Contacts	shaiqur@iastate.edu (M.S. Rahman), archanav@iastate.edu (A. Venkatachalapathy)

**Table 6:** Showing specification of videos.

Format	MP4
Video codec	H.264/AVC
Framerate	30FPS
Video bitrate	11.88Mbit/s
Resolution	1920 × 1080
Aspect ratio	16:9

### 2.1 Gaze zone

**Fig. 2:**
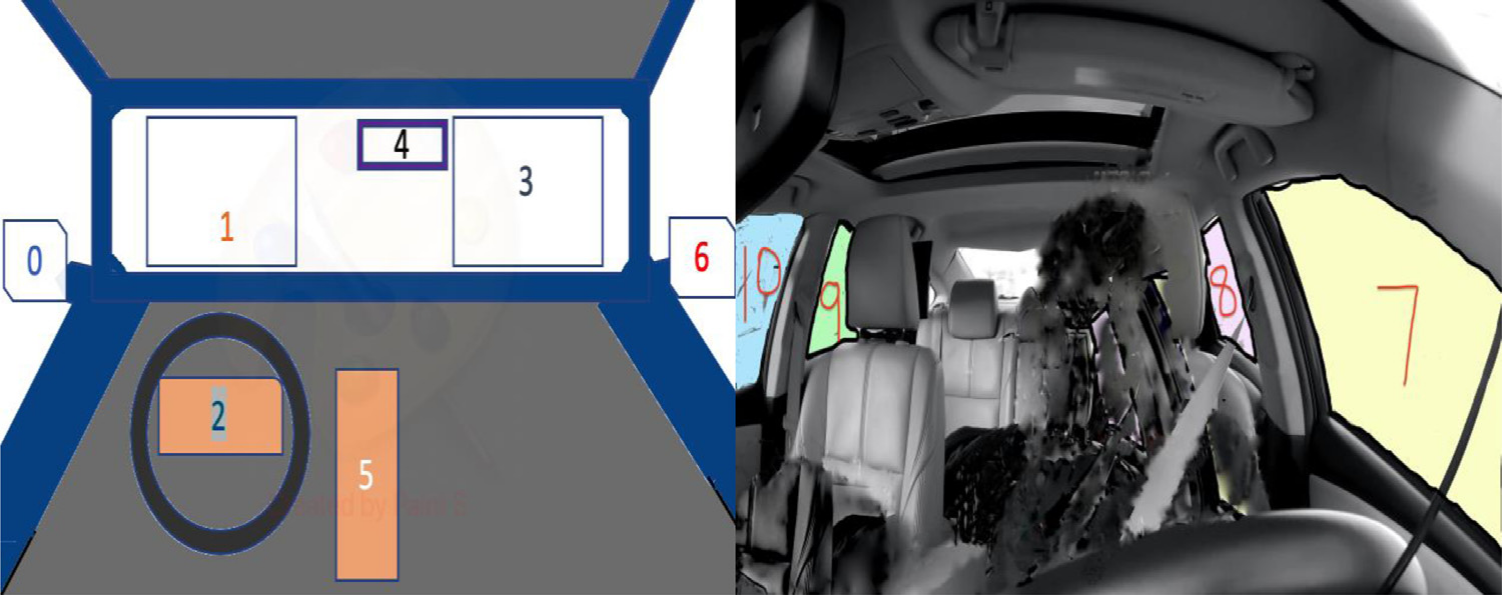
Showing the gaze zones/regions

Fig. 2 shows the eleven gaze zones in the car, and Table 7 lists all the gaze zones. The duration (up to 24 seconds) and order of activities were random for each participant.

**Table 7:** Showing gaze zones.

S. No	Gaze zones/regions
0	left-rear mirror
1	forward window
2	speedometer
3	right-frontal window
4	rear mirror
5	control panel and shift
6	right-rear mirror
7	left-side window
8	left blind spot
9	right-side blind spot
10	right-side window

### 2.2 Distracted behavior

**Fig. 3:**
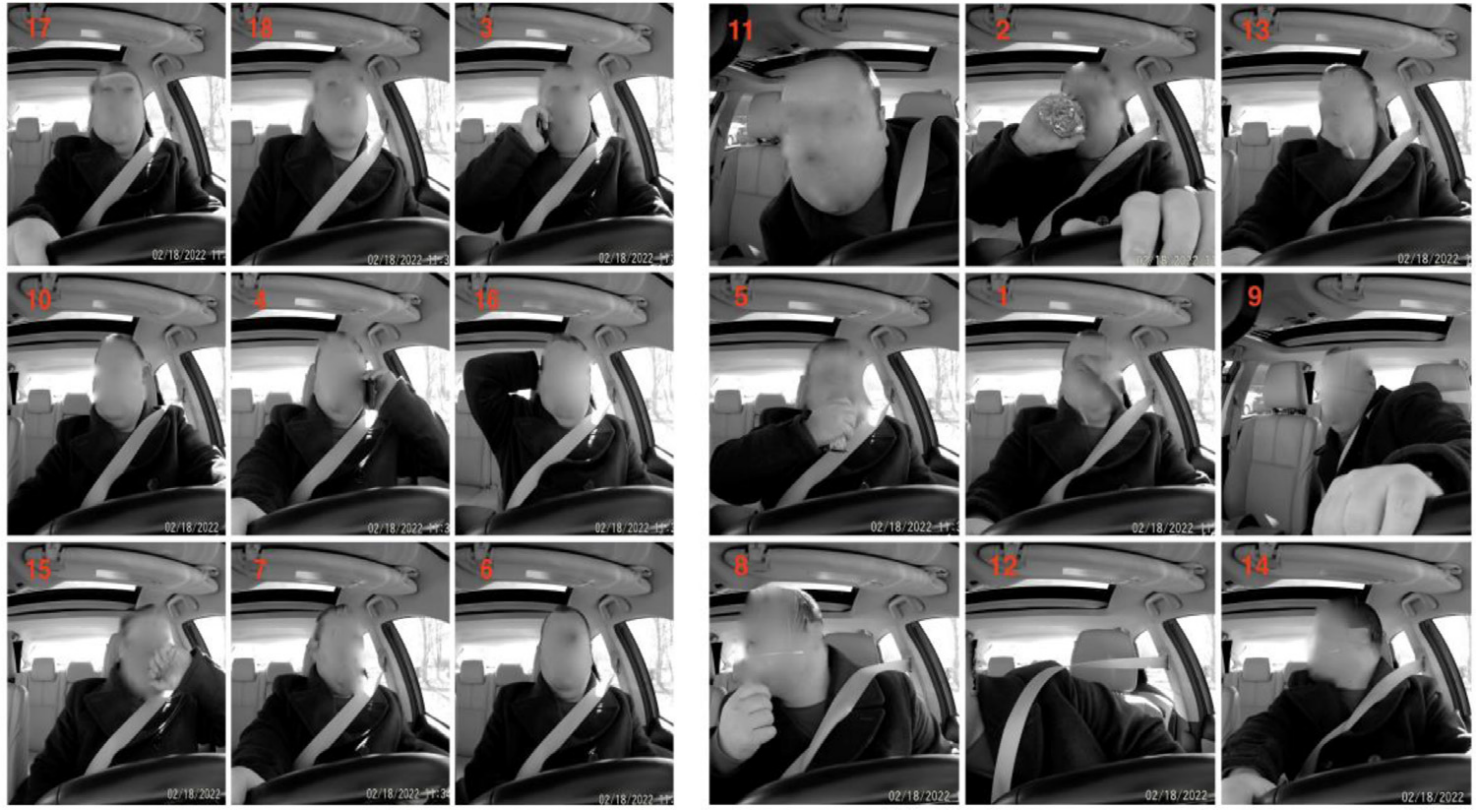
Showing distracted behaviors

Each participant continuously performed eighteen distracted driver behavior, as shown in Fig. 3, for a small-time interval. We have listed the eighteen activities in Table 8. The duration (up to 24 seconds) and order of activities were random for each participant.

**Table 8:** Showing distracted behavior.

S. No	Distracted driver behavior
1	Normal Forward Driving
2	Drinking
3	Phone Call(right)
4	Phone Call(left)
5	Eating
6	Text (Right)
7	Text (Left)
8	Hair / makeup
9	Reaching behind
10	Adjust control panel
11	Pick up from floor (Driver)
12	Pick up from floor (Passenger)
13	Talk to passenger at the right
14	Talk to passenger at backseat
15	Yawning
16	Hand on head
17	Singing with music
18	Shaking or dancing with music

### 2.3 Method

We requested each participant to follow the instructions played on a portable audio player, or we instructed them by sitting in the backseat. After a participant completed one set of activities, we requested them to repeat the set by wearing a hat or sunglasses. One set of gaze activities took approximately 5-6 minutes to complete, while the distracted driving activities took around 10 minutes. The whole set of activities took about one hour to finish.

The sequence and duration of each activity were randomized for each participant to make the data complex for analysis.

### 2.4 Instructions for activities

We created an instruction video in the English language for both activity types. For gaze activity type, the video showed the region to gaze, and for distracted activity type, it displayed activity names in the English language like drinking, eating, etc. The instructions started by explaining the kind of activity the participant would perform. Then the instruction video would play a beep sound. At that point, the participant would begin acting and continue until they heard another beep sound.

We added the beep sounds to synchronize the videos from different camera views and help annotate the activities manually.

### 2.5 Data pre-processing

By default, each camera would split the video files after a fixed time interval. As a result, each participant's raw data had multiple video files from a single camera. Hence, we combined all the video files from a single participant into a single file using python and FFmpeg [8].

“ffmpeg -f concat -safe 0 -i video-input-list.txt -c copy {out}”

We sorted the video files and added the file names in the video-input-list.txt file. Then using FFmpeg, we concatenated the videos listed in the text file giving us a single file {out}.

After that, we split the concatenated video into multiple video files based on the activity types: gaze, gaze with appearance block, distracted, and distracted with appearance block.

"ffmpeg -ss {start} -t {dur} -i {p} -c copy {out}"

Where {start} represents the start time of the activity type (gaze/distracted), {dur} represents the length of that activity type, {p} represents the path of the input file and {out} represents the output file name.

Finally, we synchronized the videos from the three camera views based on the beep sound played in the instruction.

### 2.6 Data annotation

We annotated each video from each camera for each participant manually. The annotation file includes each activity's start and end times—more information in Table 1.

## Ethics Statements

We first got approval from IRB and then we started the data collection process. We confirm that each participant has signed the IRB approved informed consent form prior to the data collection. The consent form clearly states that the data (showing the face) will be used in data challenges and competition and will be released for worldwide use as data set. For Figs. 2 and 3, we confirm that we got the IRB approved consent from the participants.

IRB committee: Institutional Review Board at Iowa State University

IRB ID: 21-462

## CRediT Author Statement

**Mohammed Shaiqur Rahman:** Writing- Original draft preparation, Data curation, Software, Methodology, Investigation. **Archana Venkatachalapathy:** Writing -Review, Data curation, Investigation. **Jiyang Wang:** Formal analysis, Study Inception and Design. **Anuj Sharma:** Study Inception and Design. **Senem Velipasalar Gursoy:** Study Inception and Design. **David Anastasiu:** Study Inception and Design. **Shuo Wang:** Study Inception and Design.

## Declaration of Competing Interest

The authors declare that they have no known competing financial interests or personal relationships that could have appeared to influence the work reported in this paper.

## Data Availability

Synthetic Distracted Driving (SynDD1) Dataset (Original data) (Mendeley Data).
